# Cyclebase 3.0: a multi-organism database on cell-cycle regulation and phenotypes

**DOI:** 10.1093/nar/gku1092

**Published:** 2014-11-05

**Authors:** Alberto Santos, Rasmus Wernersson, Lars Juhl Jensen

**Affiliations:** 1 Novo Nordisk Foundation Center for Protein Research, Faculty of Health and Medical Sciences, University of Copenhagen, 2200 Copenhagen, Denmark; 2 Center for Biological Sequence Analysis, Technical University of Denmark, 2800 Lyngby, Denmark; 3 Intomics A/S, 2800 Lyngby, Denmark

## Abstract

The eukaryotic cell division cycle is a highly regulated process that consists of a complex series of events and involves thousands of proteins. Researchers have studied the regulation of the cell cycle in several organisms, employing a wide range of high-throughput technologies, such as microarray-based mRNA expression profiling and quantitative proteomics. Due to its complexity, the cell cycle can also fail or otherwise change in many different ways if important genes are knocked out, which has been studied in several microscopy-based knockdown screens. The data from these many large-scale efforts are not easily accessed, analyzed and combined due to their inherent heterogeneity. To address this, we have created Cyclebase—available at http://www.cyclebase.org—an online database that allows users to easily visualize and download results from genome-wide cell-cycle-related experiments. In Cyclebase version 3.0, we have updated the content of the database to reflect changes to genome annotation, added new mRNA and protein expression data, and integrated cell-cycle phenotype information from high-content screens and model-organism databases. The new version of Cyclebase also features a new web interface, designed around an overview figure that summarizes all the cell-cycle-related data for a gene.

## INTRODUCTION

One of the arguably most fundamental processes to eukaryotic life is the mitotic cell cycle, the process through which a cell replicates its genetic material and divides to become two cells. This process has thus been intensely studied for decades in several model organisms, both at the molecular level and at the phenotypic level.

Today, numerous large-scale datasets related to the mitotic cell cycle exist. These include microarray-based time courses of mRNA expression (1–9), mass-spectrometry-based proteomics on protein expression during the cell cycle (10,11), systematic screens for cyclin-dependent kinase (CDK) substrates (12,13) and high-content screening for knockdown phenotypes (14–22). Together, these datasets provide a wealth of information on the mitotic cell cycle and its many regulatory layers. However, it takes great effort to collect, analyze and combine this amount of heterogeneous data, especially when it is scattered across databases and supplementary files from articles.

The aim of Cyclebase is to address exactly that problem. Earlier versions of Cyclebase primarily addressed the challenge of jointly analyzing and visualizing the many available mRNA expression time courses for a gene and to allow easy comparison across orthologous and paralogous genes. In this new version, we have greatly expanded the scope of the database to include also the results from more recent proteomics and high-content phenotype screening efforts. To accommodate these new types of data into the resource, we have completely redesigned the web interface and the underlying database architecture. The centerpiece of the new interface provides a simple overview of the complex underlying data on the cell-cycle regulation and phenotypes of a gene.

## NEW AND UPDATED DATA IN CYCLEBASE 3.0

All data for a given organism in Cyclebase is mapped onto a common set of genes. In version 3.0, we have updated these gene sets to be consistent with the latest version of the eggNOG database (23), from which we also obtain information on orthologs and paralogs.

In addition to remapping all existing microarray studies from the previous version of Cyclebase, we have incorporated one additional study for *Saccharomyces cerevisiae*, which used tiling arrays to globally measure gene expression with high temporal resolution in synchronized cell cultures (9). After inclusion of these additional time courses, we reassessed the periodicity of all *S*. *cerevisiae* genes and recalculated the time of peak expression for all genes deemed periodic.

For human genes, we have complemented the existing microarray expression data with data from two quantitative proteomics studies (10,11). Both studies used mass spectrometry to quantify protein levels in cell cultures from six different time intervals of the cell cycle, which approximately represent G1, G1/S, early S, late S, G2 and M phase. To make the two datasets as comparable to each other as possible, we represent the observed intensity value for each time interval as the intensity ratio relative to unsynchronized cells, which both studies also measured.

Post-translational regulation is at least as important as transcriptional regulation and explains much of the difference observed between transcriptomics and proteomics studies. To capture also this aspect of cell-cycle regulation, we import information on experimentally determined substrates of cell-cycle-related kinases from the latest version of the Phospho.ELM database (24). Unlike earlier versions of Cyclebase, we import information not only for members of the CDK family of kinases, but also for the Polo, Aurora, NEK and DYRK families. For *S. cerevisiae*, we also import the Cdc28p substrates identified in two high-throughput screens (12,13). For CDKs, we complement the known substrates with sequence-based predictions using a regular expression for a CDK consensus site ([S/T]P.[KR]); we opted to use this simple approach because the results were nearly identical to the highest confidence CDK sites predicted by the NetPhorest tool (25). As in earlier versions, we also include sequence-based predictions of PEST regions and D-box and KEN-box motifs, all of which suggest that the protein may be targeted for ubiquitin-mediated proteasomal degradation. As a new feature, we now also include data from two mass-spectrometry studies of a cell-cycle-related Posttranslational Modifications (PTM) closely related to ubiquitination, namely SUMOylation (26,27).

The largest addition of new data to Cyclebase v3.0, however, comes from high-throughput screens and database annotations of cell-cycle phenotypes. Recent years have seen a flood of such datasets made by automated microscopy and siRNA knockdown in human cell lines, such as the Mitocheck Project (19,20) and several other high-content screens (15–18,21). To ensure the quality of the data integrated in Cyclebase, we filtered these screens to include only phenotypes observed with at least half of the siRNAs targeting the gene in question. Large-scale cell-cycle phenotype screens also exist for *S. cerevisiae* (14) and *Schizosaccharomyces pombe* (22). However, as these screens are included in the respective model organism databases (28,29) along with phenotype annotations from many other experiments, we opted to import phenotype associations from these databases instead of the individual screens. To standardize the phenotype terminology, we made use of existing ontologies, namely the Cellular Microscopy Phenotype Ontology for the screens of human cell lines and the Ascomycete Phenotype Ontology and the Fission Yeast Phenotype Ontology (30) for the two yeasts. As these are organism-specific ontologies, we unified them using the entity–quality model to map all their cell-cycle-related terms to combinations of Gene Ontology and Phenotypic Quality Ontology terms. These common terms are the basis for the visualization of the phenotypes.

## REDESIGNED WEB INTERFACE

Until now, the main focus of Cyclebase web interface was on visualizing data from time-course microarray studies. Because of the broader scope of Cyclebase 3.0, we have completely redesigned the web interface to put less emphasis on the showing expression profiles in individual experiments and more focus on providing an overview of the heterogeneous cell-cycle data related to a gene of interest.

**Figure 1.**
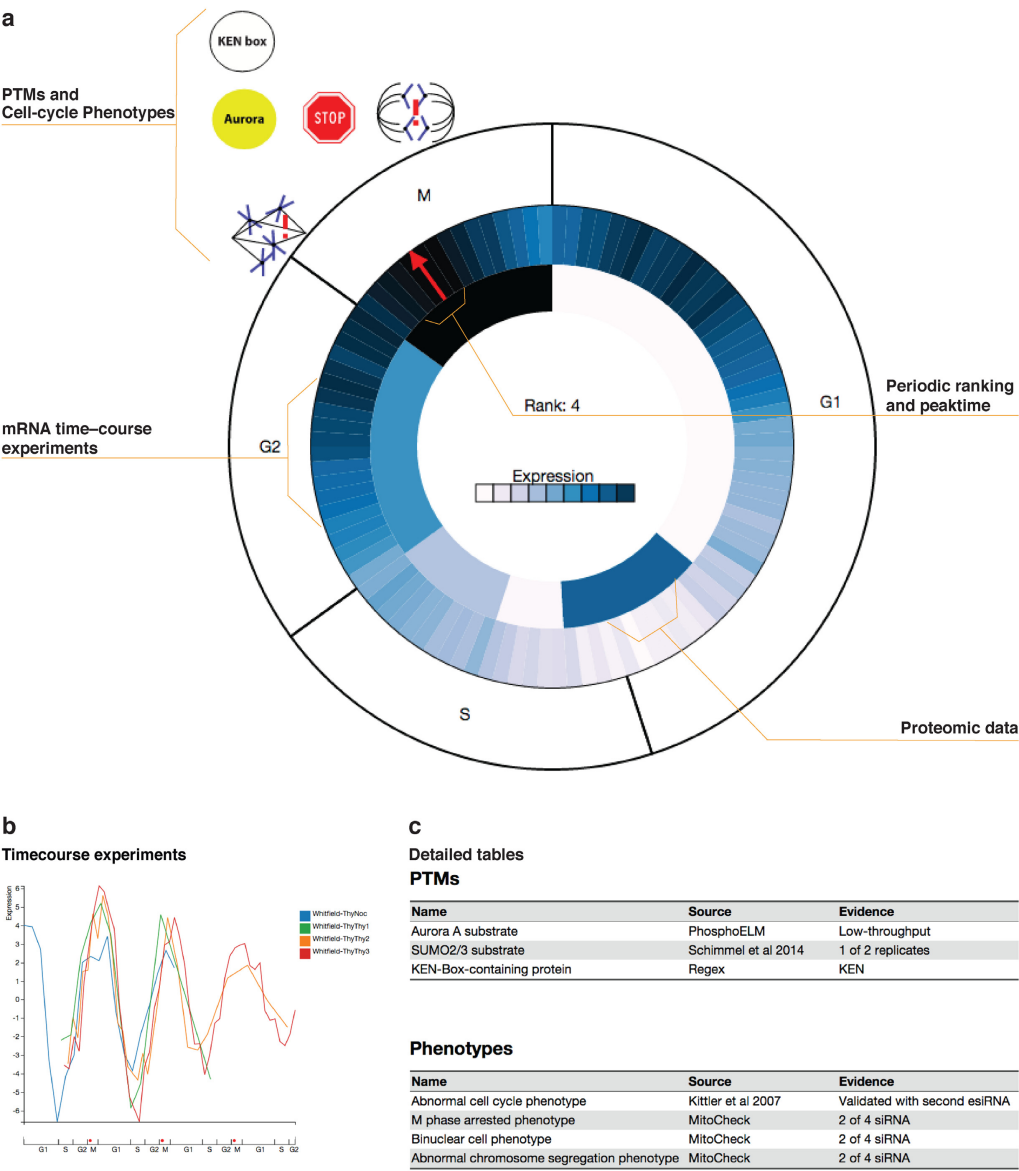
Overview of cell-cycle regulation and phenotypes. (**a**) A key feature of Cyclebase 3.0 is the new visualization, which aims to provide a concise overview of cell-cycle regulation and phenotypes for a gene. (**b**) For a more detailed view of the transcriptomic data, we normalize and align the individual time course studies, to allow all expression data for a gene to be plotted on a common time scale (percentage of cell cycle). (**c**) Further detail on PTMs, degradation signals and organism-specific phenotypes is provided in the form of tables with linkouts to the original sources whenever possible.

The central part of the new web interface is the new overview figure (Figure 1), which aims to provide an at-a-glance overview of the transcriptional and post-translational regulation during the cell cycle as well as the cell-cycle-related phenotypes. To this end, we map the information onto a schematic of the cell-cycle phases. Inside the circle representing the phases, we summarize the available expression data. We show a running average of the data from transcriptomics time courses as a circular blue scale heat map, with a red arrow designating the estimated time of peak expression. When proteomics data are available—presently only the case for human—we show these inside of the transcriptomics data. As for the latter, we show the average of the results from the available experiments; however, because the proteomics time courses are not nearly as finely time resolved, we display the average expression within each of six-phases window instead of as a running average. Outside the schematic of the phases we show icons that represent the presence of certain PTM sites and degradation signals as well as observed cell-cycle phenotypes. The icons are placed at the point in the cell cycle that they are primarily associated with. Hovering over any part of the overview figure will provide additional information in the form of tooltips.

For users who want to see further on the temporal regulation, Cyclebase 3.0 provides plots of the temporal mRNA expression profile of a gene according to each individual time-course experiment. This visualization will be familiar to users of earlier versions of Cyclebase but features improved interactivity and visual quality on modern displays. We have achieved this by reimplementing the functionality using client-side vector graphics instead of server-side bitmap graphics.

Two tables below the two visualizations provide further detail on the PTMs/degradation signals and phenotypes, respectively. In addition to what can also be seen in the overview figure, the tables show the source of the evidence and provides linkouts to the source whenever possible. The phenotype table furthermore lists additional phenotypes that are related to the cell cycle, but cannot be associated with any particular phase or transition.

The last table on the page about a gene shows its orthologs and paralogs in the organisms covered by Cyclebase. Like in the previous version of the database, the table shows the results of the analyses of transcriptomics studies for each gene. We have made this part of the table more compact so that it now only shows for each gene if it was deemed periodically expressed or not, and in which phase or at which transition its transcript level peaks. This allows us to also summarize for which of the genes cell-cycle-related phenotypes were observed and which phases these phenotypes relate to.

## PERSPECTIVES

With this major update, Cyclebase is well positioned to continue to serve the scientific community as a one-stop-shop for cell-cycle data. The scope of the database has been expanded from its initial focus on transcriptomics time courses to reflect the developments in experimental technologies, most notably proteomics and high-content screening. Having such data integrated into Cyclebase will help illuminate the complex interplay between the different layers of regulation and understand how regulation relates to the phenotypes observed when knocking down or overexpressing.
